# Hepatoprotective activity of bio-fabricated carbon quantum dots-decorated zinc oxide against carbon tetrachloride-induced liver injury in male rats

**DOI:** 10.1186/s40360-025-00924-0

**Published:** 2025-05-01

**Authors:** Fatma El-Zahraa S. Mohamed, Hebat-Allah S. Tohamy, Mohamed El-Sakhawy

**Affiliations:** 1 Nutrition & Food Science Department, Research of Food Industries and Nutrition Institute, National Research Centre, 33 El Bohouth St. (former El Tahrir St.), P.O. 12622, Dokki, Cairo, Egypt; 2 Cellulose and Paper Department, National Research Centre, 33 El Bohouth Str, P.O. 12622, Dokki, Giza, Egypt

**Keywords:** Carbon quantum dots, Liver disease, Carbon tetracholoride (CCL4), Cirrhosis, Histopathological liver

## Abstract

**Background:**

Cirrhosis is considered as a severe liver disease that causes partial liver damage as well as total liver destruction; It remains a significant health concern. Sugar cane juice is a particularly beneficial beverage, and its waste products are crucial for treating numerous illnesses. As compared to traditional treatments, zinc-doped carbon quantum dots (Zn/CQDs) are easy-to-prepare, economically invested, high nutritive value and environmentally safe substance.

**Materials & methods:**

This study investigated the hepatoprotective effects of zinc-doped carbon quantum dots (Zn/CQDs) against carbon tetrachloride (CCl₄)-induced liver injury in male Wistar albino rats. Zn/CQDs were synthesized using a microwave-assisted method and characterized using FTIR and XRD techniques. Meanwhile, a liver Cirrhosis model induced by carbon tetrachloride (CCl_4_) was utilized to determine the inhibitory effects of sugar cane juice mixed with Zn/CQDs against liver Cirrhosis. Biochemical parameters, including AST, ALT, and uric acid, were measured to assess liver function. Histopathological analysis was performed to examine liver tissue damage.

**Results:**

In this study, Zn/CQDs were extended from 1.62 to 5.45 nm. The results demonstrated that Zn/CQDs exhibited significant hepatoprotective effects by reducing liver enzyme levels and mitigating histopathological changes. However, the study also highlighted the need for further optimization of the used vehicle delivery method, such as sugarcane juice, which is showed a marginal impact on liver function. Sugar cane juice with Zn/CQDs decreased aspartate amino transferase levels (AST) and improved the uric acid concentration. It means a protection from the toxins effect by controlling the liver enzyme levels; but also, elevated levels of alanine aminotransferase (ALT) indicate ongoing liver injury. Overall, this study provides future insights into the potential of sugar cane juice with Zn/CQDs as a high nutritive value additive to drinks and food; it is investigated for plants waste as a novel green therapeutic strategy for liver diseases. Further research is necessary to explore the underlying mechanisms of action and to optimize their formulation for clinical applications.

**Conclusion:**

Overall, this study provides promising insights into the potential of Zn/CQDs as a novel green therapeutic strategy for liver diseases.

## Introduction

The alarming rise in toxic liver injury, fueled by factors like alcohol and drug abuse, has drawn urgent medical attention. Acute liver injury (ALI), a major contributor to liver failure with high mortality, is under intense scrutiny. ALI serves as a critical gateway to severe liver dysfunction, marked by complications like hepatic encephalopathy and impaired protein synthesis [1]. Limited by high costs, donor shortages, and immunosuppression risks, liver transplantation falls short as a universally accessible cure for ALI. Novel therapeutic strategies are urgently needed to fill this critical gap in treatment options [2]. At present, no effective drug for the treatment of liver fibrosis has been approved for marketing [3].

Strong hepatotoxins like carbon tetrachloride (CCl_4_) cause severe harm to the liver, leading to cirrhosis, irreversible scarring, and functional impairment. Sadly, there isn’t a treatment for this persistent illness. Ongoing research, however, attempts to create methods to lessen the negative consequences of CCl_4_ exposure and delay the development of liver damage. It has been demonstrated that nanomaterials enhance hepatic regeneration, or the liver’s capacity to heal itself [4].

Nanomaterials are poised to revolutionize healthcare by offering innovative solutions for disease diagnosis, drug delivery, and tissue regeneration [5]. By harnessing their unique properties, researchers can develop targeted therapies with enhanced efficacy and reduced side effects [6, 7]. Carbon quantum dots (CQDs) can be named as nanomaterials with particle size less than 100 nm which could be derived from diverse sources, including agricultural wastes such as sugarcane bagasse (SC) [8, 9]. One possible way to valorize these materials is to convert the SC, a major source of pollution to the environment, into CQDs [10, 11]. There are numerous ways to create CQDs from agricultural waste. One popular technique is hydrothermal synthesis, which heats the waste material with water and other chemicals in a high-pressure reactor [12]. Another method, microwave synthesis, involves heating the waste material in a microwave oven with water and other chemicals [13, 14]. The particular waste material used, and the synthesis method employed affect the properties of CQDs made from agricultural waste. Nonetheless, CQDs obtained from agricultural waste typically have advantageous chemical and optical characteristics, making them appropriate for several uses [10, 14].

In a previous study, ZnO nanoparticles added to the animal feed, in the doses studied, showed adverse effects on hematologic parameters, cytokines, oxidative stress, cytochrome enzymes, liver enzymes, and histologic parameters of rat liver [15]. In this study, we doped CQDs with Zn (Zn/CQDs) via a Microwave method. The microwave synthesis of Zn/CQDs presents a promising approach to produce these CQDs nanomaterials with potential applications in various fields [8]. This method offers several advantages over conventional synthesis techniques, including rapid synthesis times, energy efficiency, and precise control over reaction conditions. By utilizing microwave energy, the synthesis process can be accelerated, leading to significant time savings. Additionally, microwave heating can be more energy-efficient compared to traditional heating methods [8]. Furthermore, microwave synthesis allows for precise control over reaction parameters such as temperature and pressure, enabling the optimization of the synthesis process and the production of Zn/CQDs with desired properties [5]. This approach holds great potential for the development of advanced materials with tailored functionalities. Also, this research demonstrates the potential of Zn/CQDs as a promising therapeutic agent for liver injury. By mitigating liver cell regeneration, Zn/CQDs offer a novel approach to combat liver diseases. The ease of synthesis, biocompatibility, and targeted drug delivery capabilities of Zn/CQDs make them attractive for biomedical applications. Further research is needed to optimize their formulation and dosage for clinical use, but this study provides a solid foundation for developing effective and safe treatments for liver disorders.

## Materials and methods

### Materials

We purchased sugarcane bagasse (SC) from the Quena Company for Paper Industry in Egypt. Carbon tetrachloride (CCl_4_) was bought from BDH chemicals Ltd. in Poole, England, and used orally to treat liver cirrhosis. The following ingredients were utilized to create the experimental diet: cellulose was bought from the Laboratory of Rasayan, Fine Chemical Limited, Mumbai, India, and casein was acquired from Al-Ahram Laboratory Chemicals (Egypt). However, the components that made up the vitamin and salt combinations were bought from Fluka (Germany) and BDH (England), respectively. Instead of the items listed above, most of the other ingredients in the experimental diet were bought from the local market. Diamond Diagnostics, MDSS GmbH, Hannover, Germany provided the diagnostic kits used for spectrophotometric assessment of kidney function (urine, creatinine, and uric acid) and liver function (AST, ALT, albumin, and total protein). The chemicals utilized were analytical grade and didn’t require any additional purification.

### Preparation of carbon quantum dots

30 mg of Sugarcane bagasse (SC) with 70 mg of NaOH and 2400 mg of urea in 100 mL H_2_O were homogenized for 30 min. Then it was frozen in a refrigerator overnight to produce a dissolved solution. After one night the defrosted mixture was ultrasonicated for 2 min. In a home microwave, the sonicated solution was heated at 700 W for around 7 min [10, 16].

### Preparation of nitrogen doped carbon quantum dots doped with zinc (Zn/CQDs)

The as-produced CQDs were mixed with 1000 ppm ZnSO_4_ and subjected to ultrasonication for 10 min. Then, it was put in a domestic microwave for 120 s at 90 W. The obtained dry yellow powder is Zn/CQDs [17–19].

### Animals

From the National Research Centre’s animal housing, 26 male albino rats were taken (NRC, Dokki, Egypt). The weight distribution was 200 ± 150 g. The facility’s veterinarians kept an eye on and conducted independent evaluations of the animals kept in individual rooms. With the [RE (223) 23] Code Number in the Experimental Animal Research Unit, this work was done following Ain Shams University’s ethical research principles. Within the animal facility, the animals were kept in private rooms under the watchful eye of a veterinarian who conducted independent assessments [5].

### Experimental diet

The basal diet was performed according to AIN- 93 [20]. The Zn/CQDs were ground into a fine powder and administered orally at a dose of 0.4µ per day after dissolving 1 g in 10 ml of water and 10 ml of SC juice. Two weeks were spent acclimating the rats before the trial started. Ad-libitum water was introduced. Rats were fed on the following diets for 45 days after being split into four major groups: Groups 1 and 2 are the control negative and positive groups and their respective diets are (basal diet without/with 0.4 µ CCl_4_ orally), respectively. Groups 3 and 4 respective diets are (basal diet + 0.4 µ CCl_4_ orally + 1 g Zn/CQDs dissolved in 10 ml water), and (basal diet + 0.4 µ CCl_4_ orally + 1 g Zn/CQDs dissolved in 10 ml SC juice), respectively.

### Characterization

#### FTIR spectra

FTIR spectroscopy was recorded by Mattson-5000 (Unicam, Somerset, United Kingdom) employing the KBr disk method across the wavenumber range of 4000–1000 cm^− 1^.

#### XRD spectra

XRD was determined by Bruker D8 Advance X-ray diffractometer (Karlsruhe, Germany) using copper (K*α*) radiation (1.5406 Å) at a 40 kV voltage and a 40 mA current.

Both of crystallinity index Cr.I. (%) and d-spacing (nm) were determined using Eqs. (1) and (2).

1 $${\rm{Cr}}{\rm{.I}}{\rm{. }}\left( {\rm{\% }} \right){\rm{ = }}\left( {{{\rm{A}}_{\rm{C}}}{\rm{/}}{{\rm{A}}_{\rm{t}}}} \right){\rm{ x \,100}}$$

where A_C_ is the crystalline peak area and A_t_ is the total area [21].

2 $${\rm{d - spacing }}\left( {{\rm{d; nm}}} \right){\rm{ = }} \:\frac{0.9\:{\uplambda\:}}{\beta\:\:Cos\theta\:}$$

where λ is the wavelength, β and θ are full widths at half maxima and Bragg’s angle of the XRD peak, respectively [19].

### Biological study

Five days were spent acclimating the rats before the experiment started. Ad-libitum water was introduced. The following diet was given to the rats for twenty-two days after they were split into four primary groups: Group 1: given a baseline diet, this group served as the control. Group 2: The positive control group received a basal diet and 0.4 µg/kg body weight of CCl_4_ orally to induce cirrhosis [5]. Groups 3 and 4 were given the same diet composition as the positive group, but in addition, group 3 received 0.4 µg of CCl_4_ and 1 g of Zn/CQDs dissolved in 10 ml of water, while group 4 received the same nutrients dissolved in 10 ml of SC juice. Using the mentioned procedure, the weekly body weight gain and daily caloric consumption were computed [15]. After the twenty-two-day trial, rats were killed under halothane anesthesia after an overnight fast. Blood samples were taken from the hepatic portal vein; a small amount was put in a heparinized tube, and the rest was centrifuged for 20 min at 3000 rpm after being left to clot at room temperature. After the serum was thoroughly separated, it was put into sterile, tightly fitting plastic tubes and chilled at -20 °C until analysis.

#### Liver and kidney functions

The measurement of Alanine Aminotransferase (ALT) and Aminotransferase (AST) was conducted using a colorimetric endpoint method, following the guidelines provided by Diamond Diagnostics, MDSS GmbH, Hannover, Germany. According to the protocols at Diamond Diagnostics, MDSS GmbH, Hannover, Germany, urea and creatinine were measured.

### Histopathological examination

Samples of the liver, spleen, and thymus were obtained right after the rats in each group were slaughtered. According to Bancroft et al. (2016), the tissues were fixed with 10% neutral formalin after being cleaned with a normal saline solution to eliminate blood. They were then transported to Cairo University’s Faculty of Veterinary Medicine for histological analysis [22].

### Statistical analysis

Using automated SPSS software (SAS Institute, Cary, NC), the data was statistically assessed. A one-way ANOVA (Analysis of Variance) test with Duncan’s multiple range tests and p significance between different groups was used to evaluate the impact of various treatments [23].

## Results and discussion

### FTIR spectroscopy

**Fig. 1:**
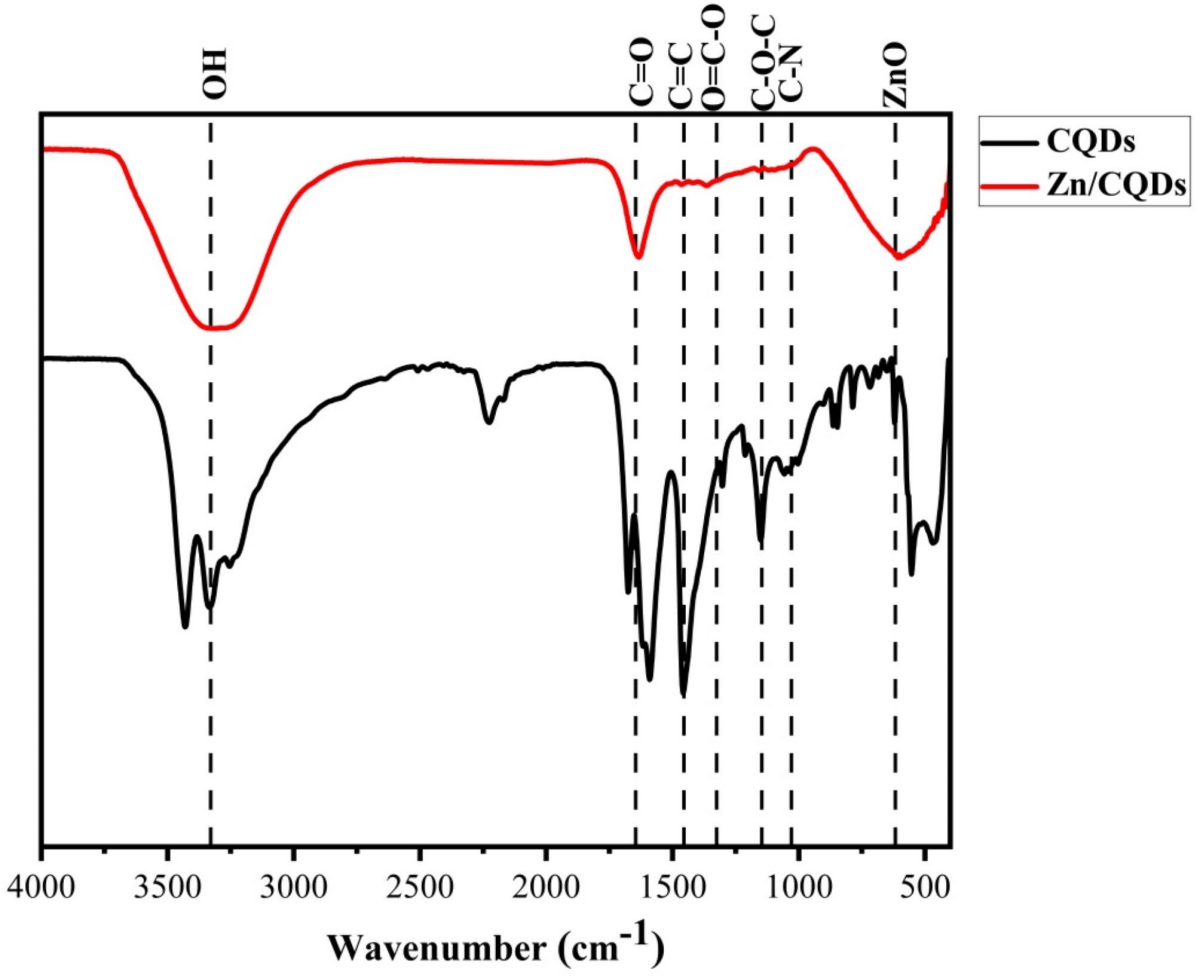
FTIR spectra of the prepared CQDs and Zn/CQDs samples

The FTIR spectra of the prepared CQDs and Zn/CQDs, as shown in Fig. 1, revealed absorption bands between 3330.66 and 3344.11 (O–H), 1457.98–1467.65 (C = C), 1301.74–1348.07 (O–C = O), 11097.36–151.34 (C–O–C) and 1047.20–1097.36 cm^− 1^ (C–N) [9, 16]. The N–H group in CQDs at 3434.75 cm^− 1^ has disappeared in Zn/CQDs, which may be due to the chelation of Zn by CQDs [19]. The decreasing of O–H intensity in the case of Zn/CQDs proves the Zn chelation. The amide peaks in CQDs at 1677.84 (amide I) and 1596.84 cm^− 1^ (amide II) are converted to one peak for Zn/CQDs at 1647.01 cm^− 1^ due to the incorporation of Zn [10, 13]. The peak at 607.50 cm^− 1^ for Zn/CQDs is related to ZnO [24].

### Morphological properties

**Fig. 2:**
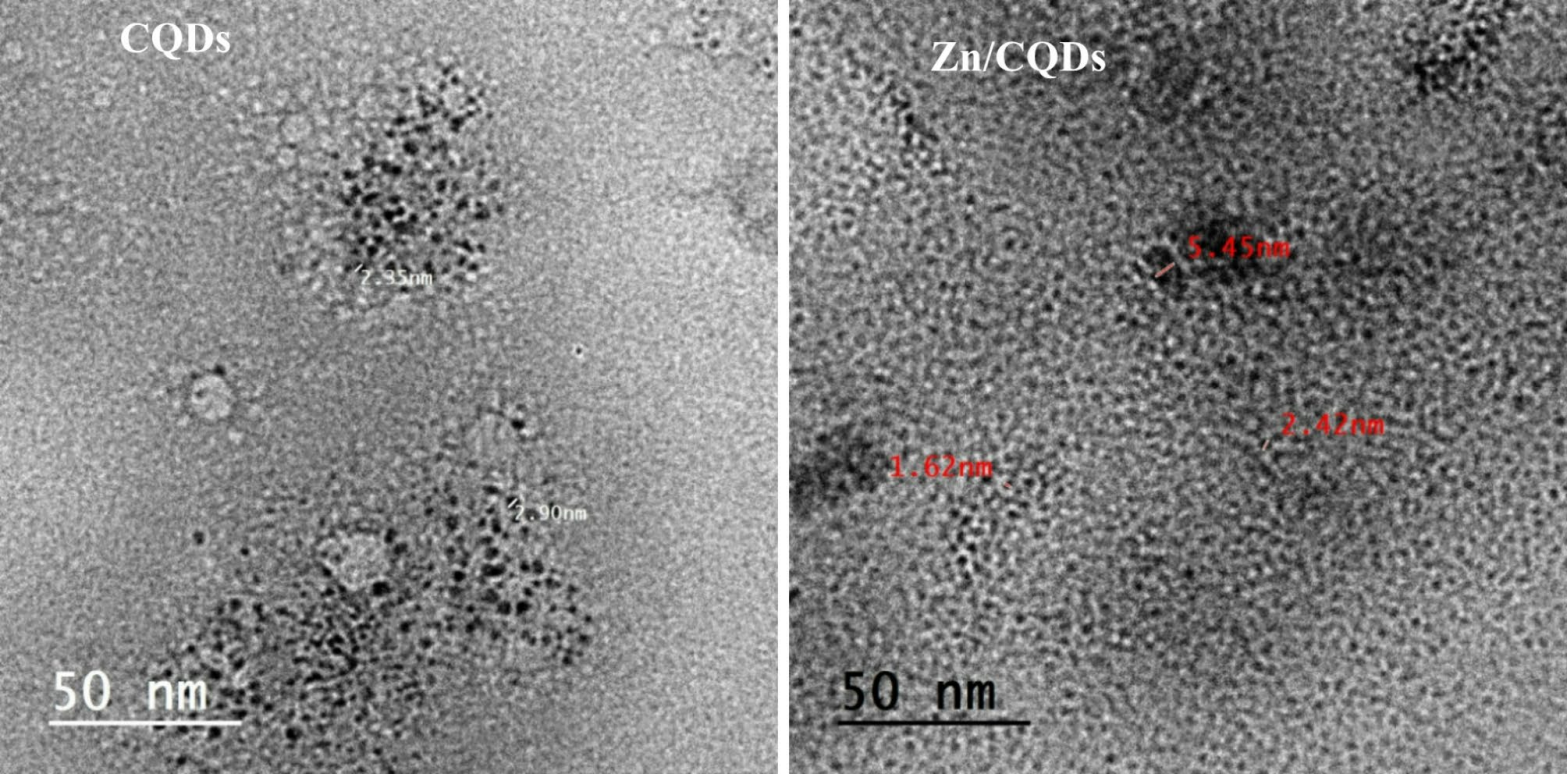
TEM of the prepared CQDs and Zn/CQDs samples

TEM images (Fig. 2) reveal that CQDs were spherical, and uniform ranged in size between 2.35 and 2.90 nm. The diameter of Zn incorporated in Zn/CQDs was between 1.62 and 5.45 nm. The small size of Zn/CQDs is due to the chemical reactions which take place on the surface of CQDs during Zn doping.

### XRD spectra

**Fig. 3:**
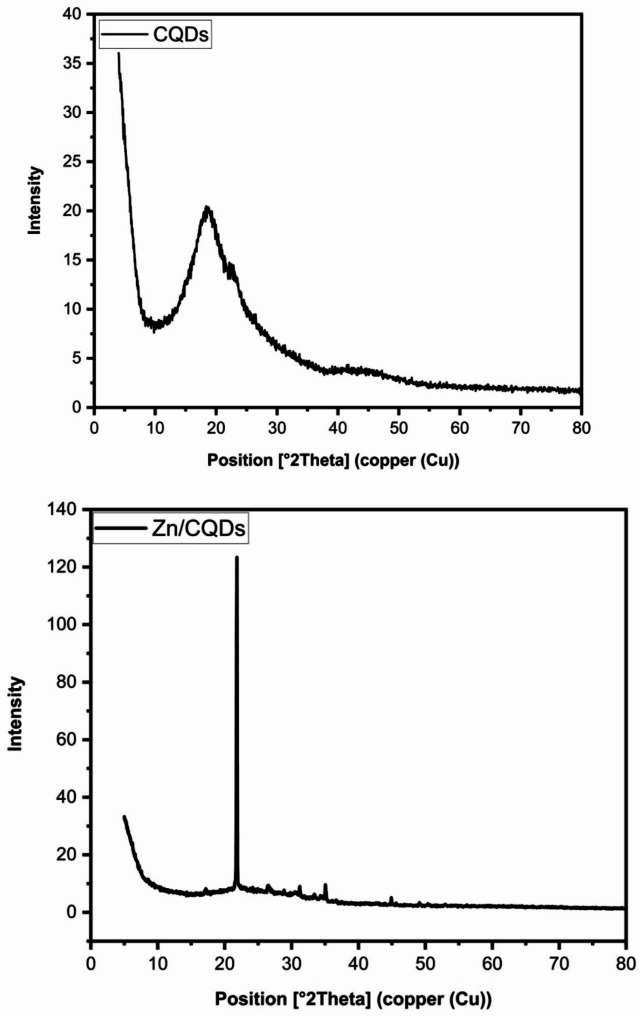
XRD of the prepared CQDs and Zn/CQDs samples

The XRD pattern, Fig. 3, revealed the known peaks of CQDs with peaks at 2θ ~ 9.0 and 17.18° related to the (001) plane, with the interlayer d spacing of 0.098 and 0.18 nm and at 2θ ~ 22.8 and 21.82° related to (002) plane due to the presence of graphene sheets in the CQDs [10].

The successful combination of Zn(II) with carboxyl groups on the surface of CQDs to form Zn-O bonds was evident. The increased d value for Zn/CQDs may be due to the incorporation of ZnO between CQDs. The peaks at 26.48, 28.88, 35.07, and 44.91° for Zn/CQDs correspond to the (002), (100), (102), and (103) crystal planes in which (002), (100) and (102) represent graphite (sp2) and (103) represents diamond (sp3) like carbon [18]. The amorphous structure of CQDs was converted to crystalline due to the incorporation of ZnO onto Zn/CQDs. This is proved from the calculated Cr.I (%) which was 44 and 91% for CQDs and Zn/CQDs, respectively.

### Biochemical analysis

Group 1: control negative group (basal diet), group 2: control positive group (basal diet + 0.4 µ CCl_4_ orally), group 3: (basal diet + 0.4 µ CCl_4_ + 1 g Zn/CQDs/10 ml H_2_O orally), group 4: (basal diet + 0.4 µ CCl_4_ + 1 g Zn/CQDs/10 ml SC juice orally). Table 1 showed a non-significant difference between all groups in food intake. But also, there was a rare significant increase in all groups compared to the control group in liver weight% & kidney weight% parameters. To assess the degree of liver dysfunction in each group AST enzyme marker levels were recorded. The percentage protection in the AST marker enzyme of group 3 was 4.68% when compared to hepatotoxic group 2. On the other hand, group 4 showed liver dysfunction. SC juice marginally impacts liver function when combined with Zn/CQDs and may pose challenges as a delivery vehicle.

On the contrary, Table 2 shows that ALT was increased for groups 3 and 4 which mean that the treatment may improve liver function, but there is still some ongoing damage to the liver cells. In other words, the treatment may target AST more than ALT, so we see a decrease in AST but not in ALT.

**Table 1:** Body weight gain, food intake, liver weight percent, and kidney weight percent for different studied rats’ group

Groups	Body weight	Food intake	Liver weight (%)	Kidney weight (%)
Group 1	9.45 ± 2.03^ab^	327.93 ± 0.25^ab^	2.96 ± 0.15^a^	0.79 ± 0.06^a^
Group 2	5.59 ± 1.11^a^	326.02 ± 0.28^ab^	4.07 ± 0.31^b^	0.92 ± 0.06^b^
Group 3	11.76 ± 1.59^b^	326.43 ± 1.15^a^	4.07 ± 0.22^b^	0.94 ± 0.04^b^
Group 4	11.60 ± 1.87^b^	328.50 ± 0.15^b^	4.08 ± 0.15^b^	0.95 ± 0.03^b^

^a^ and ^b^ are signs for grade of differences

**Table 2:** Aspartate aminotransferase (AST), Alanine aminotransferase (ALT) and uric acid for different studied rats’ group

Groups	ALT (U/L)	AST (U/L)	Uric acid (mg/dl)
Group 1	73.42 ± 7.01^a^	85.12 ± 2.25^a^	4.02 ± 0.24^b^
Group 2	161.53 ± 6.84^b^	169.21 ± 12.23^b^	3.63 ± 0.15^ab^
Group 3	186.10 ± 11.27^c^	160.15 ± 1.21^b^	3.34 ± 0.35^ab^
Group 4	186.50 ± 0.65^c^	171.75 ± 10.46^c^	3.17 ± 0.21^a^

^a^, ^b^ and ^c^ are signs for grade of differences

At the same time, the uric acid (Table 2) was decreased to 9.45 and 21.14% for groups 3 and 4, respectively, which demonstrates the effectiveness of Zn/CQDs in improving the overall function of the liver, allowing it to process uric acid more efficiently. The pharmacological effects of Zn/CQDs are investigated by the study of the biological responses to drug action. Natural product is said to exert their action by binding with cell proteins receptor [25].

### Histopathological results

**Fig. 4:**
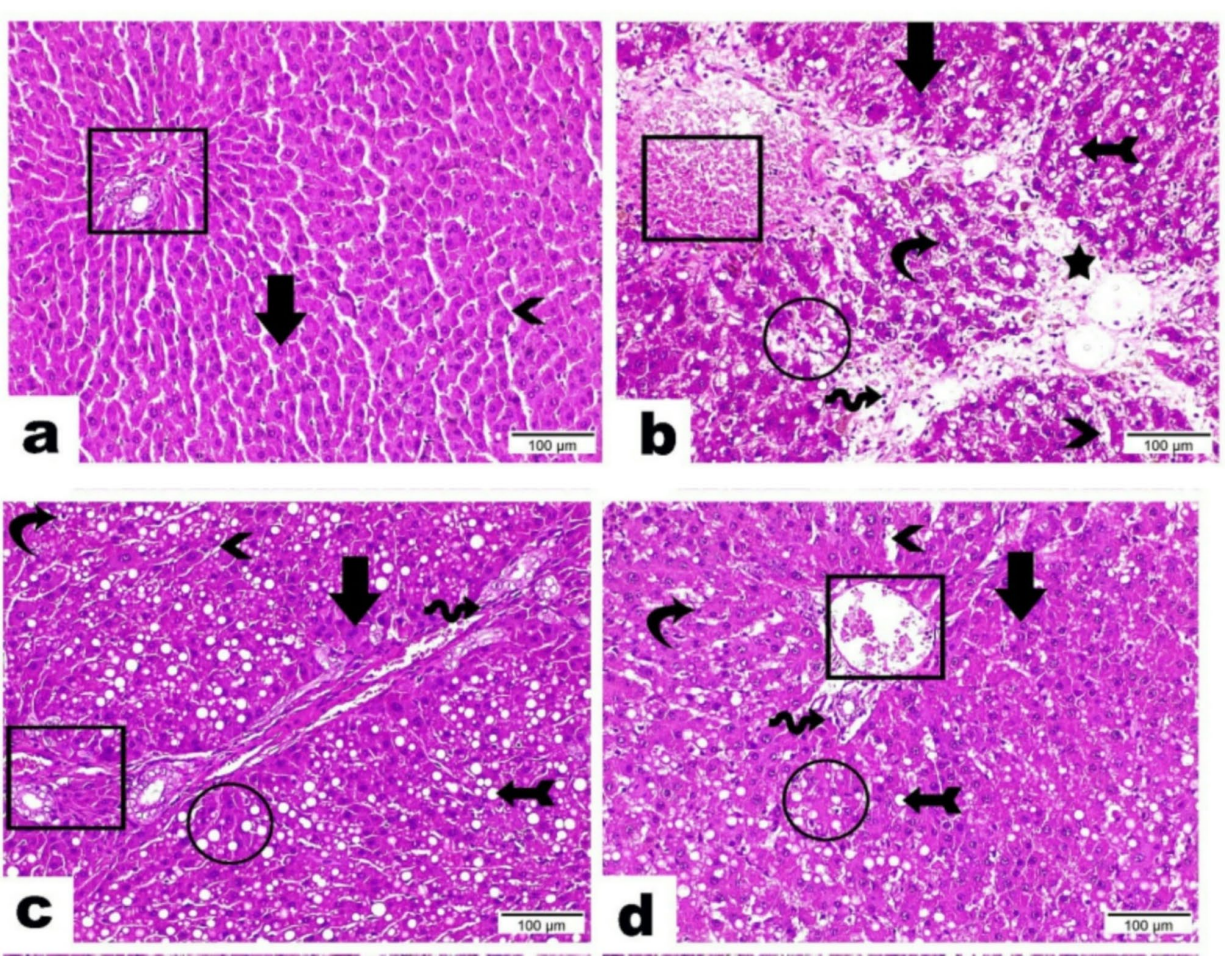
Histopathological examination of photomicrographs of the rat’s liver tissue sections belonging to different groups stained with hematoxylin and eosin: liver section of normal control rats treated with basal diet (**a**), liver section of rats treated with CCl_4_ (4 µ) alone (**b**), liver section of rats treated with Zn/CQDs (1 g/ 10 ml H_2_O) + CCl_4_ (**c**); and liver section of rats treated with Zn/CQDs (1 g/ 10 ml SC juice) + CCl_4_ (**d**)

Figure 4 for the liver section from the control negative group 1 shows the intact blood sinusoids (arrowhead), the typical architecture of the portal area (rectangle), and the hepatic cords comprising hepatocytes with central, spherical, and vesicular nuclei (central vein, arrow). The liver section from the control positive group, on the other hand, showed signs of severe hepatic injury, such as severe portal vein dilatation and congestion with degenerated endothelium (rectangle), loss of hepatic cord organization (circle), interstitial edema and fibrosis that resulted in hepatic cord dispersion (star), and inflammatory cell infiltration (wave arrow) [5]. Additionally, certain hepatocytes were found to have deep basophilic apoptotic nuclei and deep acidophilic cytoplasm (arrow), whereas other hepatocytes had hydropic degenerations (curvy arrow). Visible dilatation was seen in the blood sinusoids (arrowhead). Take note of the tail-like micro vesicular steatosis (arrow).

The liver section from group three shows signs of hepatic damage, including reduced fibrosis with few inflammatory cells (wave arrow), a regular arrangement of the hepatic cords (circle), and a portal vein that is congested but not dilated. Most hepatocytes were presented with deep acidophilic cytoplasm and an obvious increase in cytoplasmic to nuclear ratio (arrow) while few hepatocytes existed with hydropic degeneration (curvy arrow). Blood sinusoids emerged with regular structure (arrowhead). In addition, severe micro vesicular steatosis was seen (arrow with tail). Moreover, the liver Section from Group 4 exhibited a lesser hepatic degeneration evidenced by limited areas of restored cords organization (circle), mild congestion of portal vein (rectangle), the scarce number of inflammatory cells (wave arrow), few blood sinusoids with clear dilatation (arrowhead), hepatocytes detected either in intact structure (arrow) and others with deep basophilic apoptotic nuclei (curvy arrow). It was reported that bacteria treated with CQDs/ZnO displayed pronounced wrinkling and deformation, beside a visible pit, indicating the leakage and deprivation of cellular components, eventually leading to cell death [26].

## Conclusion

A facile, economical, and eco-conscious method was employed to fabricate Zn/CQDs from sugarcane agricultural residues. FTIR characterization confirmed the successful synthesis of Zn/CQDs by demonstrating the interaction between Zn and CQDs functional groups. The disappearance of the N–H peak and decreasing O–H intensity indicated Zn chelation with CQDs. The amide peaks also showed transformation due to Zn incorporation. The ZnO peak further validated the presence of Zn.

In conclusion, the study suggests that while Zn/CQDs may offer partial protection against CCl_4_-induced liver damage, as evidenced by the decrease in AST levels, it does not fully prevent liver cell injury, as indicated by the elevated ALT levels. Additionally, the decreasing of uric acid levels for both groups treated with Zn/CQDs suggests an improvement in overall liver function, potentially due to enhanced uric acid processing. However, the negative impact of sugarcane juice on liver function in group 4 highlights the importance of further investigating its interaction with Zn/CQDs and identifying safer delivery vehicles for potential therapeutic applications.

Limitations of CQDs in treating liver include; while Zn/CQDs showed a decrease in AST levels, indicating some protection against liver damage, they did not fully prevent liver cell injury as evidenced by elevated ALT levels; the study only investigated the effects in rats with CCl_4_-induced liver damage. More research is needed to determine their effectiveness in other types of liver disease and in humans; and the optimal dosage and formulation of Zn/CQDs for therapeutic applications requires further investigation.

## Data Availability

All data generated or analysed during this study are included in this published article.
